# Editorial: Culture and morality: things we value

**DOI:** 10.3389/fpsyg.2024.1528375

**Published:** 2025-01-03

**Authors:** Michael Shengtao Wu, Christine Ma-Kellams, Tian Xie, Yanyan Zhang

**Affiliations:** ^1^School of Sociology and Anthropology, Xiamen University, Xiamen, China; ^2^Department of Psychology, San Jose State University, San Jose, CA, United States; ^3^School of Journalism and Communication, Wuhan University, Wuhan, China; ^4^Department of Psychology, Jilin University, Changchun, China

**Keywords:** culture, morality, value, WEIRD bias, independent self, moral clashes

Back when the field of cultural psychology first emerged on the scene as a promising subfield within psychology several decades ago, value was the most obvious place to start (Hofstede, 2001). Of all the different ways a science could divvy up the nuances of human nature across people, places, and time, how individuals differed in their core convictions of what was important (and what wasn't) and of what was right (versus what was wrong) became powerful ways we could differentiate and understand them. People from the so-called West—or WEIRD (Western, Educated, Industrialized, Rich, and Democratic) countries at large, we now know—were individualists, concerned with the individual as a bound, stable, trait-bearing entity; those from the non-West (e.g., East Asia, South Asia, the Arab world, and Latin America) were much more collectivistic, putting group and relational concerns on par with personal ones (Henrich et al., 2010; Kitayama and Salvador, 2024).

## Rethinking WEIRD biases in morality

Historically, culture and morality were largely conceptualized from a Western perspective: the moral subject was regarded as an independent self with the right to free choice, largely unrestrained by environmental and social demands, while the socially constructed nature of the self and the common good of community members were often ignored (Sandel, 1982/1998). Moreover, as the world at large moved from rural/traditional communities to more urban/modern societies, values on individual rights and free choice—and with these, individualistic and liberal moral foundations (e.g., care and fairness)—became more prized even as they clashed against the norms of social responsibility and conservative moral foundations (like loyalty and authority), which were frequently overlooked (Greenfield, 2009; Haidt, 2007). But with the advent of cultural psychology came increasing calls for research attention to alternative cultural norms and moral practices that took into account ecological systems and temporal variations.

In recent years, a growing body of research on ecological diversity (Oishi, 2014), social class (Grossmann and Varnum, 2011), religions (Cohen et al., 2016), and social change (Varnum and Grossmann, 2017) has updated our conception of culture to expand beyond merely race or ethnicity in order to make room for additional forms of culture. A number of studies in this Research Topic “*Culture and morality: the things we value*” highlight the potent role of socioecological context (e.g., ethnicity, ideology, wealth) as macro-level predictors (Chen-Xia et al.; Grigoryev et al.; Taku and Arai; Tanaka et al.) and socioeconomic status as micro-level predictors of participants' behaviors (Hu et al.; Wu et al.; Zhang et al.), along with the role of the target being perceived (Lin et al.). Additionally, one of the most interesting aspects of culture—that also makes it more difficult to study and document—is its dynamic nature. Indeed, one of the newer topics of interest to cultural psychologists in recent years has been the issue of cultural change: what it looks like, when/where it happens, and how we can track it (e.g., Eriksson et al.).

## Cultural variance vs. universality in morality

Alongside these developments within the field of cultural psychology, moral psychology has also shifted from merely documenting cultural differences in what we value to more mechanistic questions of how and why these variations exist. Cultural researchers have long argued that morality can be divided into three major domains: community code, autonomy code, and divinity code (e.g., Kollareth and Russell, 2007; Shweder et al., 1997). The community code involves people's responsibilities within specific groups and take into account forces like social class or status, interdependence, and perceived duties or obligations among group members. In contrast, the autonomy code views individuals as independent and self-governing agents who are mainly concerned with individual rights and social justice. The divinity code pertains to sacred orders and norms derived from religion, including concerns like the maintenance of purity when it comes to both body and soul. While Western moral psychology research has historically focused on the domain of autonomy when it comes to morality, cross-cultural studies have broadened the scope of moral psychology research to include these additional domains. Since its inception, Haidt (2007) further subdivided these original three moral codes into five moral foundations: Care/Harm, Fairness/Cheating, Loyalty/Betrayal, Authority/Subversion, and Purity/Degradation (Graham et al., 2011). However, recent works on moral foundations pointed to the lack of measurement invariance of the above five-factor model, especially in non-WEIRD societies (e.g., in Iran), and proposed a six-factor model of moral foundations in which equality and proportionality, as the distinct manifestations of fairness (with the former for societal wellbeing and the latter for social order), were taken into account (Atari et al., 2020, 2023).

As an illustrative example, consider the cross-cultural research on how East Asians and European-Americans differ when it comes to basic moral foundations. Compared to Westerners who more often categorized harmful behaviors (e.g., murder, discrimination) as immoral, Chinese individuals were more likely to deem uncivilized behaviors (e.g., disrespecting parents, making loud noises, promiscuous relationships) as immoral (Buchtel et al., 2015). Similarly, Wu et al. (2011) found that Chinese participants did not consider injustice to be a problem; instead, they believed that the world was generally just and orderly, despite experiencing injustice themselves. In the face of others' suffering, Chinese participants (vs. Westerners) were less sensitive and less likely to stand up if they were not involved in some direct relationships (e.g., personal beneficiary, kinship or friendship) to the victim of unfair treatment (Wu et al., 2014; Nudelman et al., 2024). Taken together, these findings suggest that in contrast to the (presumed) universal moral norms of care and fairness that dominate morality in Western societies, Confucian cultures are more prone to take into account personal connections and face-saving as morally relevant forces.

Furthermore, research on religion and social class indicate that the morality of mental states and actions differs among different religions and socioeconomic backgrounds. For example, Cohen et al. have done several studies that demonstrate how Protestants pay more attention to thoughts about immoral actions than Jews, who instead feel that actions rather than thoughts determine one's moral standing (for a review, see: Cohen et al., 2016). In addition to these religious differences, socioeconomic differences also exist when it comes to moral judgments: relative to the working class, those from upper classes were more likely to engage in unethical behaviors and less likely to show empathy (including running red lights and aggressively driving at intersections; Kraus et al., 2010; Piff et al., 2012).

Not surprisingly, the breadth of research covered in the Research Topic reflects how far we have come and largely build on this existing literature of how culture, broadly defined, is a key force in dictating moral attitudes and beliefs. Some of this work reflect the continued effort to document existing differences between groups. This can be seen especially in response to global canon events like the COVID-19 pandemic. For example, when Taku and Arai tracked the rise of suicide ideation, they found that attitudes such as cherishing family and friends and value-congruence played a protective factor in Japan but a risk factor in the US during the COVID-19 pandemic. Along a related vein, Tanaka et al. examined differences between these two groups when it came to different forms of social support. They found that European Americans were more likely than Japanese to provide explicit support and more motivated to increase close others' self-esteem and feeling of connectedness, whereas Japanese individuals were more likely to provide attentiveness support, and were motivated by concern for their entire social groups. Likewise, class cultural dimensions like individualism-collectivism remained a potent predictor, such as predicting reactions to social norm transgressors in China vs. the U.K. (Chen-Xia et al.) and explaining the link between cultural identity and subjective wellbeing (Zhou et al.).

Interestingly, other studies in this Research Topic have found both evidence of cultural variance and universality, like when it comes to predicting people's protest responses to corrupt government practices across countries that vary in both levels of wealth and corruption (Grigoryev et al.). Still others collected data cross-culturally but did not focus on cultural differences per say and chose instead to highlight other mechanism variables like prior knowledge of a historical act of racial violence (Durham et al.). Additional studies have focused more on explanatory factors and mechanisms. Efforts to understand patriotism highlight the key role of gratitude through its impact on life satisfaction (Hu et al.). Other research covered here focus on group dynamics more broadly, including social status and group-based competition, and how it relates to observer punishment (Lin et al.) and unethical behavior (Zhu et al.), as well as the contributing roles of emotion (e.g., envy) and cognition (e.g., belief in a just world).

Although it is beyond the scope of this Research Topic, it should nevertheless be noted that morality also has physiological, biological, and evolutionary foundations that may be universal in nature. For instance, many developmental (Hamlin et al., 2010) and evolutionary (Brosnan and de Waal, 2014) psychologists believe that morality is innate in children and even primates rather than the result of rational development (Bloom, 2013; de Waal and Suchak, 2010). In recent years, neuroscientists have explored the brain mechanisms of these types of moral behaviors and the process of gene-culture co-evolution. Much of this research suggests that moral cognition and emotions are, to some extent, also physiological and biochemical reactions (Suhler and Churchland, 2011).

## Limitations and future directions

While we are proud of this Research Topic for its broad coverage of populations (e.g., Europeans, North Americans, Russians, Nigerians, Indian, and Chinese), cultural forms (e.g., race/ethnicity, social class, GDP), and research methods (e.g., cross-sectional surveys, longitudinal studies, experimental designs), there remains several limitations in this body of work on culture and morality. First, most of studies in this Research Topic were based on self-reported measures, with few studies, if any, tackling the implicit or unconscious learning of cultural norms and moralities. Second, despite the aforementioned rise of cultural and social neuroscience, none of the studies presented relied on neuroscientific methods despite their utility in providing insights into the mechanisms underlying cultural universality vs. variations. Third, studies on culture and morality often overlook the developmental trajectories over the lifespan, and the work presented here is no exception. Thus, we may be missing critical insights from childhood that contribute to the socialization of morality that may ultimately account for some of the cultural differences found in the studies presented here. Fourth, none of the existing research employed Big Data or artificial intelligence (AI) techniques, which may hold potential for analyzing large-scale behavioral patterns and predicting trends across diverse populations and species (e.g., humans vs. robots), especially in an era when an increasing number of human behaviors are shaped by social media (Van Bavel et al., 2024) and simulated by AI (Shanahan et al., 2023).

Lastly, none of the studies in this Research Topic touches upon the dark side of morality. Despite the contentious political era during which this Research Topic will be published, research on the moral clashes that drive social division, like those between liberal vs. conservative in the US (e.g., Graham et al., 2009) or those that threaten individual rights and liberties (e.g., Box-Steffensmeier et al., 2022), remain largely absent from this Research Topic.

Addressing these gaps could enhance our understanding of morality and facilitate the development of moral psychology to be more comprehensive in its theoretical and methodological frameworks. Therefore, future research should continue to tackle the WEIRD biases that have so long plagued psychology by examining culture and morality from the perspectives of diverse groups and ecological systems, taking into consideration both the long-standing forces of human evolution as well as the latest technological developments that may cast new insight into our understanding of good and evil.
